# Mother’s Parenting Stress and Marital Satisfaction During the Parenting Period: Examining the Role of Depression, Solitude, and Time Alone

**DOI:** 10.3389/fpsyg.2022.847419

**Published:** 2022-03-01

**Authors:** Simeng Dong, Qinnan Dong, Haiyan Chen, Shuai Yang

**Affiliations:** ^1^School of Economics and Management, Chongqing University of Posts and Telecommunications, Chongqing, China; ^2^Yangtze River Economic Research Center, Chongqing Technology and Business University, Chongqing, China; ^3^School of Economics, Chongqing Technology and Business University, Chongqing, China; ^4^School of Marxism Studies, Chongqing University of Posts and Telecommunications, Chongqing, China

**Keywords:** mother’s parenting stress, depression, solitude, marital satisfaction, alone time

## Abstract

This study examines the mechanism of maternal parenting stress on marital satisfaction based on the Vulnerability-Stress-Adaptation Model (VSAM), and draws on the needs theory to explore the role of alone time in marital relationships under different solitude preferences. The marital satisfaction Scale, Self-rating Depression Scale (SDS), Parenting Stress Scale (PSS), Preference for Solitude Scale (PSS), and alone time scale were used to conduct a questionnaire survey of 1,387 Chinese mothers in their parenting stage. The results found that: (1) in the overall group and the high and low solitude preference level group, depression plays a significant mediating role between parenting stress and marital satisfaction. (2) For mothers who prefer solitude, alone time can reduce the positive impact of parenting stress on depression, and but it cannot alleviate the negative impact of parenting stress and depression on marital satisfaction. (3) In the low solitude preference level group, alone time can aggravate the positive impact of parenting stress on depression and the negative impact of parenting stress on marital satisfaction.

## Introduction

In recent decades, due to rapid economic development and changes in people’s values, the divorce rate worldwide is increasing significantly. In Western society, marriage breakdown is an increasingly common phenomenon. Approximately 1 million families divorce in Europe each year, and more than 60% of divorced families have children (Rodríguez and Martínez-Aedoy Rojo, 2018). Since 2003, China’s divorce rate has gradually increased for 15 consecutive years (Zhang, 2020). According to data from the Ministry of Civil Affairs in China, the divorce rate in the country has risen from 2.8% in 2015 to 3.1% in 2020, and 40% of divorced couples have been married within 3 years (Ministry of Civil Affairs of the People’s Republic of China, 2020). In view of these facts, China’s rising divorce rate has also prompted scholars to start investigating its underlying causes.

Early studies on marriage took economic stress and work stress as the main factors affecting the quality of marriage (Krokoff et al., 1989; Conger et al., 1999). In recent years, parenting stress has been regarded as the direct source of stress for fathers and mothers (Ostberg and Hagekull, 2000), and the resulting dissatisfaction with the quality of marriage is an important reason for this relationship’s breakdown (Fincham et al., 1989). The increase in the divorce rate during parenting can be attributed to various factors. The mothers’ pressure accumulation in the process of parenting has also become a factor that cannot be ignored in the decline of marital satisfaction. Individuals with high stress tend to have a higher intention to divorce, and it even directly reduces the individual’s marital happiness index (Li et al., 2014). However, the pressure of parenting is multifaceted and includes the degree of father’s participation in parenting, the type of mother’s occupation, and family structure. In particular, in contemporary Chinese families, mothers are still the main providers of childcare, which requires significant time and energy. In addition to being under higher parental pressure, mothers have to face a huge contradiction between childcare and professional development (Cohler and Musick, 2018). Previous studies have also confirmed that the quality of a couple’s marriage will decline with the birth of a child (Glenn and Mclanahan, 1982; Cowan et al., 2000). Moreover, husbands and wives need to bear the responsibilities of both supporting their respective parents and educating their children. Whether these tasks can be successfully completed directly affects the success of the entire married life. If these expectations are not met, it may lead to a decline in the quality of the marriage and even the relationship’s end (Leonard and Roberts, 1998).

However, for many parents who have experienced the same parenting stress, the quality of their marriage did not get affected, and some even show higher family functions (Tedeschi et al., 1998; Lavee and Ben-Ari, 2008). This point is worthy of our attention; that is, is there a protective factor in the marriage relationship? Is this raising factor useful just to help parents relieve the pressure of parenting during the parenting period? What about the key resources to maintain marriage relationships? Given these questions, one of the main goals of this research is to explore restorative factors that have a positive impact on marriage quality. As Beach and Stanley (2007) proposed, current marriage research should focus on marriage relationships. There are positive factors that have a repairing effect in the process, and the role of this factor may provide more valuable theoretical significance for related research on marriage quality (Beach and Stanley, 2007).

In recent years, solitude has attracted increasing attention in the field of psychology, especially for its positive effects such as reducing overall emotional negativity, promoting mental health, integrating self, and restoring emotions (Chua and Koestner, 2008; Lay et al., 2020). The needs theory of Buchholz (1997) pointed out that it is fundamental to establish intimacy with people, but being alone is also equally important in a person’s growth needs. Long-term theoretical arguments have supported the importance of “solitude” in daily life (Coplan et al., 2018), because it entails self-exploration (Goossens, 2014) and self-renewal (Korpela and Staats, 2014), as well as important development background. Research on intimacy indicates that mature adults can spending time alone to resolve negative emotions such as anxiety caused by stress and can rebuild emotional balance and improve emotional connection with their partners (Rook, 1987).

### Mediating Model Linking Mother’s Parenting Stress, Marital Satisfaction, and Depression

Parenting stress refers to the stress caused by parents in the process of raising their children, including negative emotional experiences and states such as anxiety, fear, self-loss, and fatigue (Tachibana et al., 2012). Parenting stress is significantly related to individual mental health and happiness in life (Skok et al., 2006; Extremera et al., 2009). There is clear evidence that high parental stress can undermine personal well-being and the quality of intimate relationships (Coyne and Downey, 1991; Cohan and Bradbury, 1997; Karney and Bradbury, 1997). During childcare, mothers usually experience higher stress (Raikes and Thompson, 2005; Cousino and Hazen, 2013), and family relationships get affected by this kind of environment, which will lead to a sharp decline in marital satisfaction (Beach et al., 1998; Dong et al., 2022).

In addition, we believe that the stress of parenting will indirectly affect the marital satisfaction of women during parenting through depression. Scholars have successively proposed a number of theories to explain the impact of stress on marital relations, such as the vulnerability-stress-adaptation model (VSAM) (Karney and Bradbury, 1997), model of the impact of stress events on marital function (Bodenmann and Cina, 2006), and crisis theory (Karney and Bradbury, 1995). Among them, VSAM is mostly used in the research of marital quality and stability (Karney and Bradbury, 1997; Williamson et al., 2013).

The VSAM model points out that fluctuations in the quality of marriage relationships can be understood through partners’ existing, stable fragility (unique qualities, experiences, and dispositions of the individual that affect their ability to function effectively within the relationship), stressful events (external stressors and strains), and adaptive processes (thoughts and behaviors enacted by partners that promote relationship positivity or reduce relationship negativity). Adaptive processes represent interactions between spouses that evolve as couples respond to stress and are conceptualized as behavioral exchanges that may be positive or negative in valence (e.g., conflict management skills, partner support). In short, those couples who experience higher levels of stress, greater vulnerability, or adopt improper methods to adapt to negative events may have higher levels of negative emotions. Further, parenting stress often plays a “booster” role in the formation of anxiety, depression, and other psychological problems. This close relationship between individual stress and depression is also supported by a large number of empirical studies (Whitley et al., 1994; Bech et al., 2005; Yu et al., 2020). The accumulation of stress seriously endangers the mother’s mental health and increases the incidence of depression (Tachibana et al., 2012). Under long-term depression, mothers are more likely to have negative coping lifestyles and harmful approaches to marriage relationships, and produce over-sensitive stress responses, leading to lower marital satisfaction (Cicchetti et al., 1998; Henderson et al., 2003). Based on the above research, we propose the following hypotheses:

**H1:** Parenting stress is negatively associated with marital satisfaction.

**H2:** Parenting stress can reduce marital satisfaction by increasing depression; that is, depression plays a mediating role between parenting stress and marital satisfaction.

### Moderating Effect of Solitude

Larson defines solitude as “a state in which the individual’s consciousness is separated from the consciousness of others, and the individual has no information or emotional communication with others, that is, a state in which there is no relationship with the outside world on the psychological and behavioral level” (Larson, 1990). Some scholars regard solitude as an important factor in improving individual mental health (Maslow, 1970; Storr, 1988). For example, being alone can be used to get rid of external pressure and to reconnect with one’s own personal values and interests. Both adolescents and adults report that they feel more focused and happier after spending time with themselves. Adolescents who spent much time with themselves seem to be more likely to adapt to life than those who spend less time by themselves (Larson, 1990). Maslow found that the need for solitude is one of the characteristics of self-actualizing people. He also noticed that these subjects also show high interpersonal warmth and have deep relationships with their close friends (Maslow, 1970). However, some scholars regard solitude as social avoidance or loneliness, and associate solitude with a strong sense of loneliness and pain, emphasizing the side effects of solitude (Kashdan et al., 2006).

When explaining this difference in solitude, some scholars regard the individual’s solitude preference as a key factor, because subjective willingness may be the main factor that determines the balance between the positive and negative experiences of being alone (Galanaki, 2004). As stated in self-determined theory, self-determined solitude is often accompanied by positive emotional experiences and is positively correlated with good intimacy (Hammitt and Madden, 1989; Thomas and Azmitia, 2018). These seemingly contradictory findings about solitude reveal its complex nature; that is, the difference in the impact of solitude on individuals may be the result of the combined effect of spending time alone and solitude preference. Individuals who are more eager to be alone (with high solitude preference) need much time to meet their solitude needs. On the contrary, for individuals with low solitude preference, the increase in time can lead to negative results such as depression, anxiety, and breakdown of interpersonal relationships.

Importantly, in this study we considered the connection between solitude and emotion pointed out in previous studies. This is in accordance with Burger (1995) who put forward that individuals who desire to be alone are more likely to actively experience the beauty of solitude than individuals who do not care for it. In fact, those individuals who can effectively use their time by themselves are usually better able to recover from stressful situations, thus promoting the harmony between the self and the outside world, when solitude is often accompanied by positive emotional experience. In contrast, for individuals with low solitude preferences, a large amount of their alone time has been shown to be related to depression (Devries et al., 1992).

However, there is an unresolved issue in the current research on solitude, which is the connection between solitude and intimacy. Few studies currently directly discuss the effect of being alone on intimate relationships. One of the studies has shown that when an individual is overly stressed or has problems with intimacy, he/she may adopt solitude as a coping mechanism, in order to better adjust his/her emotions and avoid behaviors that undermine the relationship between the two parties (Chen and Zhou, 2012). Another study pointed out that solitude enables individuals to find different processes for understanding and accepting the existence of themselves and others, enhancing our ability to love others, and therefore making intimate and interpersonal relationships more effective (Moustakas, 1972). These results provide relevant theoretical support for our research on solitude’s effects on marriage relationships. Therefore, this research explores the moderating effect of solitary time on the relationship between parenting stress and marital satisfaction based on the individual’s solitude preference. Based on this, we propose the following hypotheses:

**H3a:** For parenting mothers with a high preference for solitude, time alone can alleviate the effect of parenting stress on depression and the decrease of marital satisfaction.
**H3b:** For parenting mothers who have a low preference for solitude, time alone may increase the effect of parenting stress on depression and reduce marital satisfaction.

### Overview of Studies

For women, parenting stress not only damages the mental health of mothers, but also affects the quality of marriage between couples. Under the background of low fertility rate and rising divorce rate in China, how to improve the mental health of women during child-rearing period and improve marital satisfaction has important practical significance. One of the aims of our study was to examine the mediating relationship of depression between maternal parenting stress and marital satisfaction. While being alone as part of everyday life is considered a self-reinforcing function, little is known about the differences between individuals with different preferences for solitude. Therefore, based on the individual’s solitude preference, exploring the moderating role of alone time in the mediating pathway is our second research purpose.

## Materials and Methods

### Participants and Procedures

The data used in this study came from a 4-month field survey from July to November 2021. Six psychology undergraduates and postgraduates had received training as research assistants, and they were mainly responsible for data collection. Participants were determined based on the residential area of the researcher and research assistant. We recruited a total of 1,473 parenting mothers in the southwest part of China (including Chongqing, Chengdu, and Yibin). We invited mothers to fill in the scales for childcare stress, depression, marital satisfaction, time alone, and preference for solitude. To ensure the accuracy of the research results, we screened out 4 severe depression samples, and eliminated 82 missing and unmatched data. A total of 1,387 valid questionnaires for mothers were obtained. The mothers were aged between 21 and 36 years (M = 26.498, SD = 2.258), children were between 0 and 6 years old (M = 3.217 SD = 1.722). The Socioeconomic Status (SES) for these samples is between 1 and 5 (M = 2.650 SD = 0.775). To ensure the applicability of the study as much as possible, we actively accommodated the participants’ time. Therefore, a part of the questionnaire was answered through the online platform.

This study was approved by the ethics committee of the first author’s university. All participants obtained informed consent. They were informed that their participation was voluntary and that they could terminate participation anytime they wanted. Participants received no rewards for their participation and were told that the data would be used for academic purposes only and would be kept confidential.

### Measures

#### Parenting Stress

The parenting stress of mothers was measured using the parenting stress scale (PSS) compiled by Berry and Jones (1995), which was translated into Chinese by Cheung (2000). There are 18 questions on the scale, including “I feel overwhelmed by my responsibilities as a parent,” “having children deprives me of many choices and autonomy in my life,” and “I sometimes worry about whether I’m doing enough for my children.” This scale uses a 6-point scale, with 1 representing “strongly disagree” and 6 representing “strongly agree.” The higher the score, the greater the parenting stress. In the present study, the Cronbach’s alpha for the scale was 0.914.

#### Depression

Regarding depression, we used the Self-Rating Depression Scale (SDS) compiled by Zung et al. (1965). The scale has a total of 20 items, including “I feel depressed,” “I feel tired for no reason,” and other questions, and uses a 4-level self-rating scale, including never, sometimes, often, and continuous. The scale is calculated by adding up the scores of 20 items, then multiplying by 1.25 and rounding up to get the standard score. According to the results of the Chinese norm, the cut-off value of the SDS standard score is 53 points, of which 53–62 are classified as mild depression, 63–72 are classified as moderate depression, and more than 73 are classified as severe depression (Zhu et al., 2021). In this study, the scale’s Cronbach’s alpha was 0.865.

#### Marital Satisfaction

For this variable, the marital satisfaction test of David H. Olson (2000) was adopted, with a total of 10 items, including “I am very satisfied with the responsibilities of both spouses in marriage,” “I am very satisfied with our leisure activities and the spouses spent together Time,” and others. The Chinese version of the scale has been validated (Zhang et al., 2021). This scale uses a 5-point score, ranging from “strongly disagree” (1) to “strongly agree” (5). The higher the total score of the scale, the higher the marital satisfaction. This scale’s Cronbach’s alpha was 0.797.

#### Preference for Solitude

The Solitude Preference Scale (PSS) was created by Burger, an American psychologist, in 1995. It is specifically used to measure a person’s solitude preference. The scale contains 12 items, and subjects are required to make choices by means of forced selection. Each item provides two mutually exclusive options, such as “I enjoy being with people/I enjoy being alone,” “I like to go to places with a lot of people and activities/I like to be in quiet places with few people,” and so on. Participants are asked to choose the one that best suits their situation from the two options. If they choose items related to solitude, 1 point is scored. The higher the score, the higher the preference for solitude. The Chinese version of this scale was translated and revised by Chen. (2012). The revised scale has high reliability. The Cronbach’s alpha for the scale was 0.711.

In order to calculate the mother’s solitude preference, we calculated the total score of each respondent’s solitude preference scale and separated the high and low solitude preference by the mean. We introduced the dummy variable “Solitude Preference” to express the mother’s solitude preference. When the total score of the solitude preference scale is greater than the average value, it is recorded as high solitude preference, and the “Solitude Preference” is assigned a value of 1, which, otherwise, is 0.

#### Time Spent Alone

Regarding for spent time alone, participants answered two questions related to being alone last week. In this question, we referred to Burger (1995) and others’ definition of solitude, which is, “to be alone or to do something alone—not including sleeping” (Burger, 1995; Coplan et al., 2019). The first question asked how much times they were alone in the last week for a period lasting at least 15 min (from 1 = “Not at all” to 6 = “more than 3 times a day”). The second asked how many total hours they spent alone in the last week [from 1 = “< 1 h (< 15 min per day)” to 6 = “More than 15 h (more than 2 h per day)].” These two results together constitute the time spent alone score for the previous week. In this study, the scale’s Cronbach’s alpha was 0.716.

The reverse scoring of the above scale has been forwarded. In addition, we conducted a variance inflation factor (VIF) test, and after the VIF test, the VIF value of each variable was far below the critical value of 10, indicating that there is no serious multicollinearity between the regression models.

### Statistical Analyses

In the present study, statistical analyses were conducted using SPSS 22.0. Data processing included the following steps. First, we calculated the descriptive statistics for the variables and then the Pearson Correlation Coefficient among these variables. Second, in order to explore the different results that may depend on different solitude preferences, we divided the samples into the high solitude preference group and the low solitude preference group based on the mean value of the preference for solitude scale, and the overall group was used as a benchmark regression in mediating model. Third, we used the PROCESS macro for SPSS (Model 4) to investigate the mediating effect of depression in the relationship between parenting stress and marital satisfaction (Hayes, 2013). Finally, in the high and low group of preference solitude, we used the PROCESS Model 59 to investigate the moderating effect of time spent alone in the direct pathway and some indirect pathway between parenting stress and marital satisfaction. Besides, child age and family SES (socioeconomic status) as the covariate were included in the mediation and moderation analyses.

The bias-corrected percentile bootstrap method based on 5,000 samples and 95% confidence intervals (95% CIs) was applied to determine whether the indirect effect was significant at the 0.05 level. All variables were standardized before testing for the mediating and moderating effects.

## Results

### Bivariate Analyses

The descriptive statistics and Pearson correlation coefficients were presented in Table 1. The results showed that mothers who scored high levels of marriage satisfaction were more likely to have low levels of parenting stress (*b* = −0.294, *p* < 0.01) and depression (*b* = −0.345, *p* < 0.01). Besides, parenting stress was positively associated with depression (*b* = 0.340, *p* < 0.01). As mentioned before, time spent alone may have different effects on people with different solitude preference. Therefore, from the overall sample, the correlation between time spent alone and other variables will become very unstable, so we will not discuss too much here.

**TABLE 1:** Descriptive statistics and correlation analysis.

	Mean	SD	1	2	3	4	5
1. Marital satisfaction	3.901	0.663	1				
2. Parenting Stress	3.137	0.791	−0.294**	1			
3. Depression	35.872	8.489	−0.345**	0.340**	1		
4. Alone Time	2.972	1.040	0.053*	–0.035	–0.015	1	
5. Solitude Preference	5.146	2.904	0.036	–0.003	–0.032	–0.021	1

**Correlation is significant at the 0.01 level (2-tailed).

*Correlation is significant at the 0.05 level (2-tailed).

*SD, standard deviation.*

### Testing for the Mediating Effect of Depression

Hypothesis 1 predicted that parenting stress would decrease marriage satisfaction, and Hypothesis 2 predicted that depression would play a mediating role in the relationship between parenting stress and marriage satisfaction. We used Model 4 of the PROCESS macro to examine these hypothesis (Hayes, 2013), and the results were presented in Table 2.

**TABLE 2:** Testing the mediation effect of depression.

Group	Overall Group (*n* = 1387)		High solitude preference (*n* = 578)		Low solitude preference (*n* = 809)	
			
	Depression	Marriage satisfaction	Depression	Marriage satisfaction	Depression	Marriage satisfaction
Parenting stress	0.340 *** (0.025)	−0.199*** (0.026)	0.375*** (0.039)	−0.284*** (0.040)	0.312*** (0.034)	−0.132*** (0.035)
Depression		−0.277*** (0.026)		−0.296*** (0.040)		−0.260*** (0.035)
Child age	−0.008 (0.015)	−0.003 (0.014)	−0.020 (0.022)	−0.009 (0.021)	0.003 (0.020)	−0.001 (0.020)
SES	0.001 (0.033)	−0.004 (0.032)	0.012 (0.049)	0.013 (0.046)	−0.006 (0.044)	−0.021 (0.044)
R^2^	0.116	0.154	0.144	0.230	0.097	0.107
F	60.523***	63.006***	42.820***	30.722***	28.813***	24.102***

***P < 0.001.

The results of all groups showed that parenting stress was positively associated with depression (βoverall = 0.340, *p* < 0.001; βhigh = 0.375, *p* < 0.001; βlow = 0.312, *p* < 0.001). Next, when parenting stress and depression are regressed to marriage satisfaction, the three groups all show similar results. Specifically, parenting stress has a significant negative correlation with marriage satisfaction (βoverall = −0.199, *p* < 0.001; βhigh = −0.284, *p* < 0.001; βlow = −0.132, *p* < 0.001), so Hypothesis 1 has supported. Further, depression has a significant negative correlation with marriage satisfaction (βoverall = −0.277, *p* < 0.001; βhigh = −0.296, *p* < 0.001; βlow = −0.260, *p* < 0.001). Furthermore, the results of bias-corrected percentile bootstrap method has shown that the 95% BootCI in each group does not contain zero [95% BootCIoverall = (−0.118, −0.072); 95% BootCIhigh = (−0.152, −0.074); 95% BootCIlow = (−0.112, −0.053)]. This result further confirmed that depression has a significant mediating effect in each group. So Hypothesis 2 has supported.

### Testing for the Moderating Effect of Time Alone

Hypothesis 3a and 3b predict that under different solitude preference, time alone would moderate the direct and some indirect pathway between parenting stress and marital satisfaction, and the results of the moderating might be different. We used Model 59 of PROCESS macro to examine this hypothesis (Hayes, 2013). The results of regression were presented in Table 3, and we also conducted simple slope tests to plot the results.

**TABLE 3:** Testing the moderating effect of alone time.

Group	High solitude preference (*n* = 578)		Low solitude preference (*n* = 809)	
		
	Depression	Marriage satisfaction	Depression	Marriage satisfaction
Parenting stress	0.313*** (0.041)	−0.245*** (0.042)	0.290*** (0.034)	−0.140*** (0.036)
Depression		−0.289*** (0.040)		−0.236*** (0.036)
Alone Time	−0.189*** (0.042)	0.113*** (0.041)	0.138*** (0.034)	−0.052 (0.035)
Int1	−0.084* (0.035)	0.009 (0.038)	0.112*** (0.031)	−0.099** (0.032)
Int2		−0.068 (0.036)		0.026 (0.032)
Child age	−0.019 (0.022)	−0.011 (0.021)	0.006 (0.019)	−0.004 (0.019)
SES	0.017 (0.048)	0.008 (0.046)	0.002 (0.044)	−0.024 (0.044)
R^2^	0.178	0.246	0.128	0.119
F	24.751***	26.559***	23.519***	15.523***

***P < 0.001; **P < 0.01; *P < 0.05; Int1: Alone time × Parenting stress; Int2: Alone time × Depression.

**FIGURE 1:**
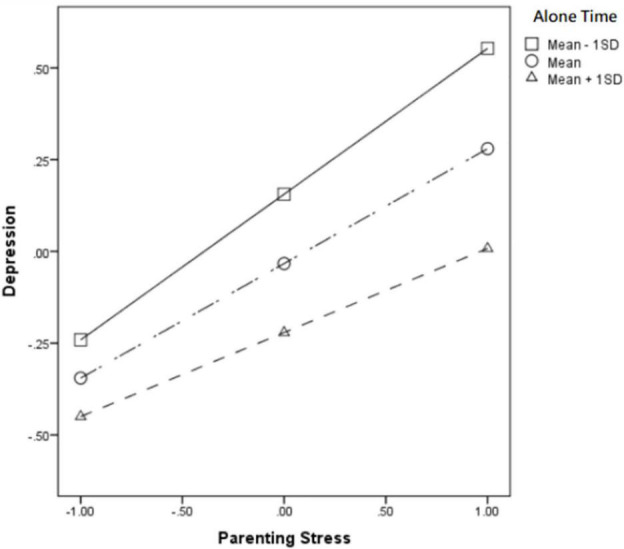
Alone time moderated the relationship between parenting stress and depression in the high solitude preference group.

In the High solitude preference level group, time alone significantly moderated the relationship between parenting stress and depression (β = −0.189, *p* < 0.05). According to a simple slope calculation, it can be seen that mothers with a higher preference for being solitude when she have much time to solitude (Mean + 1SD), the positive effect of parenting stress on depression (βsimple = 0.299) is significantly weaker than that of mothers who have less time alone (Mean − 1SD) (βsimple = 0.397) (see Figure 1). But in the path of parenting stress and depression’s influence on marriage satisfaction, time spent alone did not show a significant moderating effect. Further, according to Bootstrap test, the mediating effect of depression was −0.082 [95%BootCI = (−0.121, −0.043)] when alone time was high (Mean + 1SD), and it was −0.088 [95%BootCI = (−0.151, −0.035)] when alone time was low (Mean − 1SD). So Hypothesis 3a has supported.

**FIGURE 2:**
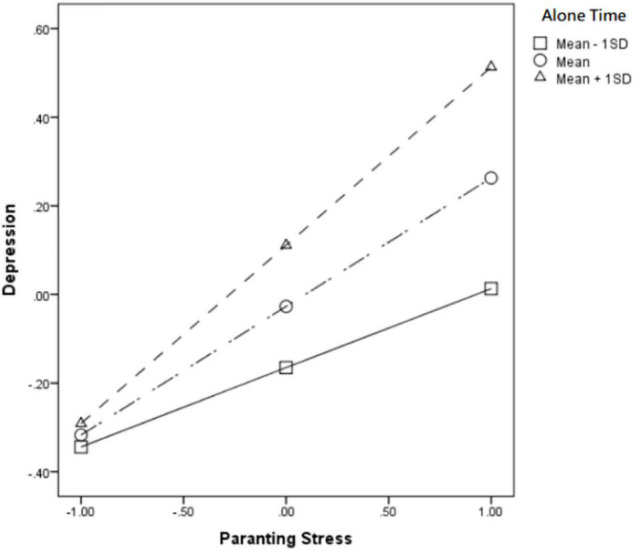
Alone time moderated the relationship between parenting stress and depression in the low solitude preference group.

**FIGURE 3:**
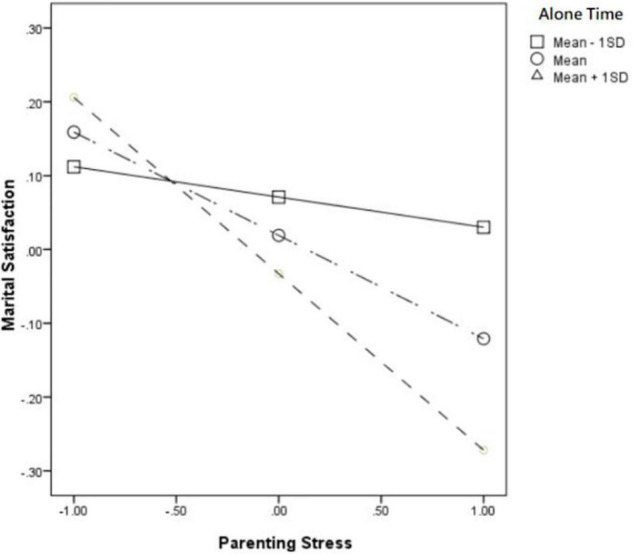
Alone time moderated the relationship between parenting stress and marriage satisfaction in the low solitude preference group.

In addition, in the Low solitude preference level group, time alone also significantly moderated the relationship between parenting stress and depression (β = 0.112, *p* < 000). For mothers with low solitude preference level, after having much time alone (Mean + 1SD), the positive effect of parenting stress on depression (βsimple = 0.402) was significantly stronger than that of mothers who having less time alone (Mean − 1SD) (βsimple = 0.178) (see Figure 2). Further speaking, time alone also significantly moderated the relationship between parenting stress and marriage satisfaction (β = −0.099, *p* < 0.01), that is to say, mothers with lower solitude preference spending much time alone (Mean + 1SD), the negative impact of parenting stress on marriage satisfaction (βsimple = −0.239) is higher than that of mothers who have less time alone (Mean − 1SD) (βsimple = −0.041) (see Figure 3). Further, according to Bootstrap’s test results, the mediating effect of depression was −0.084 [95%BootCI = (−0.134, −0.043)] when alone time was higher (Mean + 1SD), while it was lower at alone time (Mean − 1SD) is −0.047 [95%BootCI = (−0.083, −0.012)]. Further, according to Bootstrap test, the mediating effect of depression was −0.084 [95%BootCI = (−0.134, −0.043)] when alone time was high (Mean + 1SD), and it was −0.047 [95%BootCI = (−0.083, −0.012)] when alone time was low (Mean − 1SD). So Hypothesis 3b has supported.

## Discussion

Women bear the pressure of the parenting period, which is usually accompanied by lower marital satisfaction. In the context of the gradual decline in China’s fertility rate and the gradual increase in divorce rate, how to improve the mental health of women in parenting period and improve their marital satisfaction bears great practical significance. Many scholars have carried out meaningful discussions on this proposition, but the mechanism of parental stress and its effects on marital satisfaction has not been fully developed. This study first used the mediation model to explore the mechanism of depression and related influence on parenting stress and marital satisfaction. In addition, recent studies on the relationship between husbands and wives consider solitude as a trick to enhance couples’ emotions and improve marital satisfaction. However, because each person’s perception of solitude is different, passive solitude may actually cause harm in certain cases. Therefore, when a husband tries to improve the satisfaction of the other party’s marriage by giving his wife much time to be alone, he must understand the wife’s solitude preference as a prerequisite. Based on this, our research further explores the regulating effect of solitary time on women’s psychological processes after stressful situations. Overall, this study clarified a moderating mediation model to understand the impact of female parenting stress on their marital satisfaction. This mode of adjustment and mediation makes it possible to formulate targeted prevention and intervention plans to improve women’s mental and marital health when faced with the stress of parenting.

### The Mediating Effect of Depression

First, we found that mothers in parenting period may experience increased depression and decreased marital satisfaction in a stressful environment. And depression has a mediating effect in the relationship between parenting stress and marital satisfaction. Due to changes in hormone levels in the body, some women experience depression after giving birth (Brummelte and Galea, 2010). However, this understanding often leads us to ignore the importance of postpartum depression in women and to believe that this psychological change will gradually disappear with the rebalancing of hormone levels. However, this study confirms that in the parenting period following childbirth, the parenting stress that new mothers will face may cause their depression to aggravate as well, if simply attributed to hormone levels, which may cause us to neglect that other factors that possibly caused depression. For instance, due to the traditional division of labor in the family and the professionalization of modern women, during the parenting period, mothers get pressure from both life and work, such as “work-family conflict,” “personal-family conflict,” and “parenting stress.” The depression caused by these pressures makes mothers more likely to have negative cognitive styles about life and interpersonal relationships, and produce excessively sensitive or stressful reactions, which further leads to lower marital satisfaction (Cicchetti et al., 1998; Henderson et al., 2003).

### The Moderating Effect of Solitude

Another important goal of this research is to investigate the influence of solitude on mothers’ psychological and emotional changes based on the mother’s preference to be solitude. Studies have found that, under different solitude preference, time alone has a moderating effect. This effect is reflected in two paths: one is the direct path of parenting stress and marital satisfaction; the other is the indirect path of depression between them. As hypothesized, there are significant differences in the moderating effect of time alone on mothers with different solitude preferences. Specifically, in the high solitude preference group, much spending time alone alleviated the positive effect of stress on depression; while in the low solitude preference group, much spending time alone increased the positive effect of parenting stress on depression and the negative effect of parenting stress on marital satisfaction.

For mothers with a high preference to be solitude, active solitude is beneficial to alleviate the negative impact on their physical and mental health after being under pressure during the parenting period. This result is consistent with Fiske’s (1980) view on solitude and loneliness. Fiske (1980) believes that the possibility of undisturbed freedom and concentration when an individual is alone provides a unique opportunity to examine a person’s current stress and life situation. This view believes that being by oneself provides an opportunity to assess and relieve stress, and its effect can be comparable to the role of social support (Cohen and Hoberman, 1983). In addition, according to the “quality-stress interaction model,” whether an individual has psychological disorders such as depression and anxiety after encountering a negative life event mainly depends on two factors, namely, the attributes of the event itself and the subject’s psychological susceptibility. Ideal solitude has been proven to help individuals’ self-exploration (Goossens, 2014) and self-renewal (Korpela and Staats, 2014). At the same time, individuals may seek solitude in order to escape or relax, cultivate emotional renewal, and arouse positive emotions and self-awareness more (Larson et al., 1982; Larson, 1990; Burger, 1995; Lay et al., 2020). Therefore, mothers with higher levels of solitude can reduce their psychological susceptibility through active solitude after gaining much spending time alone, thereby reducing their negative reactions to stressful events. In addition, we found that the relationship between parenting stress and marital satisfaction is not significantly moderated by spending time alone. It may be because the evaluation of marital satisfaction focuses on the intimate relationship between the husband and wife, while being alone is more a process of self-exploration and renewal.

For mothers who have low solitude preference, passive solitude will aggravate their negative psychological changes after being stressed. Previous studies have confirmed that for individuals, too much time alone may be related to depression (deVries et al., 1987), and may also increase individual negative emotions and feelings of loneliness (Larson et al., 1985; Chui et al., 2014; Nguyen et al., 2017; Lay et al., 2020). In a marriage relationship, excessive passive loneliness may make mothers feel alienated and neglected by their husbands. At the same time, the boredom of being alone can also lead to the accumulation of negative emotions. Therefore, for mothers with lower propensity to be alone, more involuntary solitude during the parenting process will make parenting mothers with lower willingness to be alone become more frustrated after stress, and at the same time lead to lower marriage satisfaction. In addition, Chinese children generally enter a full-time kindergarten at the age of 3, and the time for mothers to take care of their children will be greatly reduced, and the opportunities and time for mothers to be alone will also be increased. However, for mothers who have a low preference for being alone, on the one hand, the sudden increase in spending time alone needs to adapt. On the other hand, spending less time with children may also produce feelings of emptiness and loss, which may aggravate negative psychological reactions after stress. Therefore, the mental health of mothers at this stage needs to be paid attention to.

In summary, the results of this study are significant in understanding the role of parental stress and solitude experience in the mental health of Chinese mothers, as well as the effects on the quality of their marriage. It is inevitable for mothers to endure pressure during childbirth. Therefore, it is fundamental to find ways to reduce mothers’ depression levels and increase marital satisfaction after such a stressful experience. In addition, it is particularly important for mothers to use positive coping styles to deal with stress of various nature. If family members cannot provide enough support, mothers who lack time for themselves are more likely to fall into the endless childrearing-work chores and find no way of self-realization and meaning in life. However, for individuals with a low preference for solitude, family members should provide more active verbal and action support. Through companionship and communication, they can help mothers get out of stressful dilemmas, and guide mothers away from negative stress coping styles. This is to help them ease their burdens during parenting. In case of stressors, the promotion of mental health and healthy marital relationships is essential.

## Conclusion

The results of this study reveal the internal mechanism and consequent effects of parental stress on marital satisfaction. First, we examined the mediating role of depression in the relationship between mothers’ parenting stress and marital satisfaction. Second, we explored the moderating effect of time alone on the relationship between parenting stress, depression, and marital satisfaction for mothers with different solitude preferences. The results of the study show that depression has a significant mediating effect between mother’s parenting stress and marital satisfaction. In addition, for mothers with a high preference for solitude, time alone can buffer the negative impact of parenting stress on depression. However, for mothers with a low preference for solitude, the increase in spending time alone aggravated the impact of parenting stress on depression and reduced marital satisfaction. Therefore, mothers may have higher depressive symptoms when under parenting stress, leading to lower marital satisfaction.

### Limitations and Further Research Directions

The current study has several limitations that warrant further consideration. First, the data in this study were obtained from a cross-sectional survey, so causality cannot be established. Meanwhile, one of the goals of this study was to understand the impact of maternal stress during parenting on marital satisfaction and protective factors in marriage. However, our analysis is not comprehensive enough and does not extend to the whole country. In the future, a large-scale, in-depth longitudinal study of parents in child-rearing period is needed, especially parents in different child-rearing periods, to explore the difference in the impact of parental stress on marriage quality, so as to test the applicability of this study’s results. Second, the factors that influence parenting stress are extremely complex. In addition to the mother’s own factors, work pressure, economic situation, stressful life events, and other factors will also affect the psychology of both parents and, to a certain extent, affect the parenting stress. Therefore, future studies need to separate the overlapping effects of parental stress and other potential stressors in the parents’ lives. Finally, our results show the influence of solitude preference and time alone. However, in addition to the solitude preference, the type of solitude, personality differences, and other factors also affect the quality of marriage. Therefore, we need further in-depth research in the future.

## Data Availability Statement

The raw data supporting the conclusions of this article will be made available by the authors, without undue reservation.

## Ethics Statement

The studies involving human participants were reviewed and approved by Ethics Committee of Chongqing University of Posts and Telecommunications. The patients/participants provided their written informed consent to participate in this study.

## Author Contributions

SD contributed to the experimental design and execution of the study, modification of the experimental design, and data analysis and guided the writing of the manuscript. QD and HC completed data analysis and wrote the first draft of the manuscript. SY participated in the experimental design and analysis of experimental results. All authors contributed to revision, read, and approved the submitted version.

## Conflict of Interest

The authors declare that the research was conducted in the absence of any commercial or financial relationships that could be construed as a potential conflict of interest.

## Publisher’s Note

All claims expressed in this article are solely those of the authors and do not necessarily represent those of their affiliated organizations, or those of the publisher, the editors and the reviewers. Any product that may be evaluated in this article, or claim that may be made by its manufacturer, is not guaranteed or endorsed by the publisher.
